# 

**Published:** 1906-06-23

**Authors:** 


CONTENTS.

**Comparative Medicine** - - - - - - - - - 203
**The Prevention of Trade Accidents** - - - - - 204
**Annotations** - - - - - - - - - - - 205
**Medical Opinion and Movement** - - - - - - 206
**Hospital Clinics—**
Fractures in the Vicinity of the Elbow-joint - - - - - 207
Cerebral Symptoms and Hæmoptysis in Pneumonia - - - 211
**Tinned and Preserved Foods** - - - - - - - 211
**Remedies and their Uses** - - - - - - - - 214
**The Book World of Medicine and Science** - - - 215
**Medical Appointments and Vacancies** - - - 215
**Hospital Administration—**
Current Hospital Topics - - - - - - - - 216
King Edward VII. Sanatorium - - - - - - - 217
Metropolitan Hospital Sunday Fund - - - - - - 218
Hospital Meetings - - - - - - - - - - 219
**Editor's Letter-Box** - - - - - - - - - 220

*NURSING SECTION.*

**Notes on News from the Nursing World—** The Army Service and Efficiency—The Colonial Nursing Association Staff—Exoneration of the Queen's Nurse—Duty and Discipline—Local Probationers—The Local Government Board and Trivial Disputes—Queen's Nurses and the Rates—An Australian Matron and Her Nurses—Imperial Military Nursing Service—The Heywood-Johnstone Memorial Home—Training Midwives in Monmouthshire—The District Nurse and the Care of Infants—An Unregistered Midwife Accused of Murder—Short Items - - - - - - - - - - - 169-172
**The Nursing Outlook** - - - - - - - - 173
**The Care and Nursing of the Insane** - - - - 174
**The Nurse's Clinic** - - - - - - - - - 175
**Incidents in a Sister's Life** - - - - - - - 176
**The Government and Registration** - - - - - 177
**Lord Elgin on the Colonial Nursing Association** 177
**The Promotion of the Higher Training of Midwives** - - - - - - - - - - - 178
**London Biblewomen and Nurses Mission: the Jubilee** - - - - - - - - - - - 179
**Everybody's Opinion** - - - - - - - - 179
**Appointments** - - - - - - - - - - 180
**Novelties for Nurses** - - - - - - - - 180
**A Book and Its Story** - - - - - - - - 181
**Notes and Queries** - - - - - - - - - 182
**For Reading to the Sick** - - - - - - - 182

